# Tumor-Associated Macrophages in Oncolytic Virotherapy: Friend or Foe?

**DOI:** 10.3390/biomedicines4030013

**Published:** 2016-07-07

**Authors:** Nicholas L. Denton, Chun-Yu Chen, Thomas R. Scott, Timothy P. Cripe

**Affiliations:** Center for Childhood Cancer and Blood Diseases, Division of Hematology/Oncology/Blood and Marrow Transplant, Nationwide Children’s Hospital, The Ohio State University, Columbus, OH 43205, USA; Nicholas.Denton@nationwidechildrens.org (N.L.D.); Chun-Yu.Chen@nationwidechildrens.org (C.-Y.C.); trscott1@crimson.ua.edu (T.R.S.)

**Keywords:** oncolytic virus, macrophage, carcinoma, neuroblastoma, sarcoma

## Abstract

Cancer therapy remains a challenge due to toxicity limitations of chemotherapy and radiation therapy. Oncolytic viruses that selectively replicate and destroy cancer cells are of increasing interest. In addition to direct cell lysis, these vectors stimulate an anti-tumor immune response. A key regulator of tumor immunity is the tumor-associated macrophage population. Macrophages can either support oncolytic virus therapy through pro-inflammatory stimulation of the anti-tumor response at the cost of hindering direct oncolysis or through immunosuppressive protection of virus replication at the cost of hindering the anti-tumor immune response. Despite similarities in macrophage interaction between adult and pediatric tumors and the abundance of research supporting macrophage modulation in adult tumors, there are few studies investigating macrophage modulation in pediatric cancers or modulation of immunotherapy. We review the current state of knowledge regarding macrophages in cancers and their influence on oncolytic virotherapy.

## 1. Introduction

Cancer was the cause of over half a million deaths in the United States (US) in the year 2013 alone [1]. Cancer is the leading cause of disease-related death in US children and is projected to catch up to heart disease in becoming the leading cause of death in US adults as well [1]. The standard therapies for treating solid tumors including surgery, chemotherapy, and radiation therapy are only partly effective in treating patients with aggressive tumors and cause short-term and long-term toxicities [2,3,4]. Therefore, there is urgent demand for more tumor-selective therapies that can treat aggressive tumors while remaining safe for cancer patients.

One approach to cancer therapy that has gained significant traction recently is the use of oncolytic viruses that selectively replicate in and destroy cancer cells. These oncolytic viruses are capable of destroying tumors not only through their direct infection and oncolysis of tumor cells, but also by activating the host’s anti-tumor immune response [4,5,6,7,8]. While tumor inflammation is known to inhibit viral replication via an interferon response and therefore may impede direct oncolysis of tumor cells, some cancers respond to a virus-induced activation of an anti-tumor immune response [9,10]. A clinical example is melanoma injected with Talimogene laherparepvec (T-Vec, trade name Imlygic; Amgen, Thousand Oaks, CA, USA), which is now approved by the US Food and Drug Administration (FDA) as a therapy [11,12,13]. T-Vec is attenuated by deletions of the genes encoding ICP34.5, a neurovirulence factor, and was designed to promote anti-tumor immunity by expressing human Granulocyte-Macrophage Colony Stimulating Factor (GM-CSF). To increase chances of tumor antigen presentation, the virus is also deleted for ICP47, a protein that blocks peptide loading onto the major histocompatibility complex (MHC). In a phase III trial, over three quarters of lesions directly injected with virus shrank. Although anti-tumor immunity was not directly measured, the fact that over half of the uninjected skin and nearly a third of the visceral lesions also responded suggests an immunologic effect. Other oncolytic viruses are being designed for anti-tumor immune stimulation using other immune-stimulatory molecules [14]. Despite its success, there is still a need to understand and circumvent the barriers to successful virotherapy because not all patients or injected lesions respond to treatment.

One of the key regulators of cancer immunotherapy is the tumor-associated macrophage (TAM) population [15,16,17]. Traditionally, macrophages are activated towards one of two polarization states: the classically activated M1 pro-inflammatory macrophage or the alternatively activated M2 immunosuppressive macrophage [17,18,19,20,21,22,23,24]. Classically activated M1 macrophages induced by interferon γ (IFNγ) and lipopolysaccharides are considered anti-tumor macrophages due to the expression of inducible nitric oxide synthase and the secretion of cytotoxic reactive oxygen species and pro-inflammatory cytokines [17,22,23,24]. Inflammatory macrophages attract natural killer (NK) cells and dendritic cells (DCs) to the tumor site through expression of chemokines CCL20, CXCL10 and CXCL11 while macrophage secretion of IFNα and IL-12 activates NK cells and DCs; activated NK cells and DCs in turn secrete IFNγ, interleukin (IL)-12 and IL-15 which promote pro-inflammatory macrophage, NK cell, and DC activation [4]. In addition to innate immune responses, M1-type macrophages attract and stimulate T cells through secretion of CCL15, CCL20, CXCL9, CXCL10 and CXCL11 [4]. Alternatively, macrophages activated by IL-4 are considered pro-tumorigenic due to the expression of growth-promoting, pro-angiogenic, and extracellular matrix remodeling signals via vascular endothelial growth factor (VEGF), IL-8, matrix metalloproteinase (MMP)-9, transforming growth factor β (TGFβ) and T cell suppression molecules [17,18,19,20,21,22,23,24]. These so-called M2 macrophages are also associated with a non-immune-stimulatory phagocytosis of apoptotic cancer cells known as efferocytosis [17]. Recently, it has become clear there are intermediate states and macrophages cannot always be classified so distinctly, and terms such as “M2a”, “M2b”, etc., “regulatory macrophage” and “tumor-associated macrophage” have been used to define multiple activation states, causing difficulties in research replicability. To harmonize across research groups, some have proposed labeling macrophage subtypes based on factors to which they have been exposed or molecules they express [21,22]. Regardless, it is clear that tumor macrophage activation states can greatly influence the efficacy of tumor immunotherapy.

The role of tumor macrophages in oncolytic virus therapy is understudied and potentially controversial in both adult and pediatric cancers. On the one hand, M1-like macrophages are predicted to enhance virus-mediated activation of the anti-tumor immune response; however, M1-like macrophages may also promote an anti-viral immune response with early clearance of virus. M2-like macrophages are associated with tumor angiogenesis, metastasis, and suppression of the anti-tumor immune response; however, M2-like macrophages may also suppress the anti-viral immune response and promote oncolysis. Here we review evidence gathered so far on the role of macrophages in modulating tumorigenesis and the anti-tumor efficacy of oncolytic virotherapy, summarized in Table 1, to help determine when macrophages are “friend” or “foe”.

## 2. Macrophages, Prognosis, and Immunotherapy

The presence of high levels of tumor-associated macrophages is associated with a poor prognosis in patients with breast, prostate, bladder, cervical, ovarian, lung, brain, and skin cancers [22,23,48,52]. This finding makes sense, given the known functions of M2-like tumor macrophages. In the context of some immunotherapies, however, macrophages may be favorable and contribute to stimulation of the anti-tumor immunity [10]. Macrophages in the subcapsular sinus also have anti-tumor functions in blocking extratumoral vesicles containing immunosuppressive cytokines from reaching the tumor-draining lymph node, and then assist in presenting tumor-associated antigens to the lymphoid cells and activating adaptive immunity to tumor-associated antigens [20,21,53]. Tumor macrophages are also vital for tumor-specific antibody therapy–mediated efficacy [54]. Investigators have also used macrophages as cell carriers for targeted delivery of oncolytic viruses into the tumor microenvironment [55].

Osteosarcoma is the most common bone tumor, with an overall survival of 70% but a <30% survival for those with metastatic disease. Osteosarcoma is replete with macrophages that express IL-10, which inhibits the T-cell IFNγ expression necessary to maintain active T cell immunotherapy [14,56,57]. IL-10 signaling from immunosuppressive macrophages also induces PD1 and TIM-3 expression in T cells, resulting in negative regulation of IFNγ and TNFα, leading to exhaustion and/or apoptosis of T cells [20,21,22,56]. Osteosarcoma tumor macrophages also produce high motility group box 1 (HMGB1), which interacts with the receptor for advanced glycation end products (RAGE), toll‑like receptors, and CD24, resulting in tumor proliferation, invasion and angiogenesis mediated by NFκB and VEGF signaling [58]. Osteosarcoma secretion of MCP-1 attracts TAMs while stimulation from Th2 cytokines IL-4 and IL-13 induces pSTAT3 activation of M2 TAMs [59]. These findings suggest macrophage regulation may also be important in immunotherapy for osteosarcoma.

Ewing sarcoma is the second most common bone-derived tumor in pediatrics with an overall survival of 70%, with only <30% surviving metastatic or recurrent tumors. These cancers harbor a characteristic chromosomal translocation that creates a hybrid gene between EWS and a member of the ETS gene family, most commonly FLI1 [23]. The poor prognosis appears to be caused in part by macrophage influence. Tumor macrophage infiltration is abundant and correlated with poor prognosis in Ewing sarcoma patients, similarly to adult tumors [23,25,26,27]. EWS/FLI1 suppresses miRNAs such as let-7a, which normally suppresses transcriptional targets of STAT3, MMP-2 and CCND-2, leading to increased macrophage infiltration, tumor proliferation, and metastasis [25]. VEGF is involved in tumor angiogenesis and metastasis and is also upregulated by EWS/FLI1 and by tumor macrophages expressing IL-1 and MCP-1 [23,28]. In a positive feedback loop, MCP-1 can also be expressed by Ewing sarcoma xenograft tumors, leading to more macrophage recruitment and angiogenesis associated with IL-8 secretion [29]. Depletion of tumor macrophages with liposome-encapsulated bisphosphonates or their inhibition with the small molecule anti-inflammatory drug Samapimod inhibited tumor proliferation and invasion in vivo [22,27]. In addition to immunosuppressive macrophage activation, Ewing sarcoma also expresses macrophage colony stimulating factor (M-CSF), TNFβ, VEGF, and receptor activator of nuclear factor kappa-B ligand (RANKL) which differentiate tumor macrophages into pro-tumorigenic M2-like osteoclasts [22,27,28,29,30,31,32,33,34]. Overall, Ewing sarcoma EWS/FLI1 and MCP-1 expression promotes tumor development through immunosuppressive macrophage and M2-like osteoclast activation.

Neuroblastoma is the most common solid tumor outside of the brain in children. MYCN-amplified neuroblastoma is particularly difficult to treat, attributed in part to the immunosuppressive tumor macrophage inhibition of DCs, NK cells, and T cells [24,35]. Neuroblastoma has decreased expression of miR-451, which inhibits macrophage migration inhibitory factor (MIF), resulting in increased macrophage infiltration [36]. There is also evidence that immunosuppressive macrophages (CD206/CD163) induce the cyclo-oxygenase–prostaglandin E2 pathway in aggressive MYCN+ neuroblastoma stromal cells, contributing to proliferation, invasion, angiogenesis, chemotherapy resistance, and immunosuppression [22,37]. In a preclinical model, an M-CSF blockade with the small inhibitor molecule BLZ945 decreased immunosuppressive macrophage (CD14/CD206) activation and increased inflammatory macrophage (HLA-DR/CD86) activation as well as DC and T cell infiltration, resulting in inhibition of tumor progression [35]. While it is difficult to generalize, these mechanisms of immunosuppressive macrophage activation and the resulting resistance to immunotherapy appear to be shared by adult and pediatric cancers.

## 3. The Tumor Macrophage Influence on Oncolytic Virus Efficacy

Colorectal cancer models generally benefit from inflammatory macrophage activation in combination with oncolytic virus therapy. In preclinical studies, oncolytic poxvirus vvDD-CCL11 enhanced immunogenic programmed necrosis in colorectal cancer; the anti-tumor adaptive immune response correlated with increased IFNy expression following virus infection [38]. Another oncolytic vaccinia virus, GLV-1h68, was associated with increased NK and macrophage infiltration and increased levels of many pro-inflammatory cytokines and chemokines involved in both antiviral and anti-tumor immune response (GCP-2, KC/GROα, IFNγ, CXCL10, IL-3, IL-6, Lymphotactin, M-CSF1, MIP-1β, MCP-1, MCP-3, MCP-5, RANTES) [39]. Aside from activation of the anti-tumor immune response, inflammatory macrophages also express macrophage metallelastase, which has anti-angiogenic effects and improved oncolytic adenovirus spread in colorectal tumors [40]. While the presence of inflammatory macrophages has been shown to benefit colorectal and some pediatric cancers, their friend role in response to oncolytic viruses in pediatric tumors has not yet been studied.

Glioblastoma multiforme is the most common and lethal brain tumor in adults. In contrast to studies in other tumor types, oncolytic virus therapy efficacy for glioblastoma is improved with the suppression of the innate immune response. This suggests that glioblastoma anti-tumor efficacy is more reliant on direct oncolysis than inflammatory stimulation. While activation of inflammatory macrophages (CD86/LYSC) in other tumors improved OV anti-tumor efficacy, inflammatory macrophage activation inhibited oncolytic herpes simplex virus (oHSV) anti-tumor efficacy in glioma in part through the TNFβ-mediated inhibition of virus replication [41,42]. Activation of Cysteine-rich 61 protein (CCN1) also inhibited the oncolytic virus anti-tumor efficacy in glioblastoma through activation and infiltration of inflammatory TAMs and NK cells expressing IL-1β, IFNγ, CXCL10, and MCP-1/3 [43,44]. Antibody neutralization of CCN1 improved the glioma response to oncolytic virus therapy most dramatically in glioblastoma models with high macrophage infiltration [43,44]. The activation of immunosuppressive macrophages with TGFβ was also shown to inhibit innate NK cells, inflammatory macrophages, and microglia, resulting in increased virus replication and oHSV anti-tumor efficacy in glioblastoma [45].

In models of breast cancer, macrophage infiltration correlates with poor patient prognosis, but there is a mixed response in terms of the influence of macrophages on oncolytic viruses. Oncolytic paramyxoviruses’ (measles/mumps) anti-tumor efficacy was enhanced by human monocyte-derived macrophages, independent of the initial macrophage polarization state and virus replication [46]. However, the inhibition of TGFβ in breast cancer bone metastases with an oncolytic adenovirus-expressing soluble TGFβ receptor II fused with human immunoglobulin Fc fragment reduced M2-like osteoclast activity and tumor progression [47]. In a study using vesicular stomatitis virus (VSV), macrophage secretion of interferons activated JAK/STAT anti-viral pathways in the cancer cells; IFN-α/β antibodies or a JAK inhibitor diminished this anti-viral response and improved VSV anti-tumor efficacy [48].

Pancreatic cancer also has a mixed response with macrophage-induced inflammation or immunosuppression in oncolytic virus therapy. While TGFβ-expressing macrophage infiltration correlates with poor prognosis in pancreatic carcinomas, the epithelial-mesenchymal transition by E-cadherin displacement of TGFβ stimulation exposes Nectin-1, which increases oHSV entry in pancreatic cancer cells [41]. High TGFβRII expression on pancreatic carcinomas also provides a selective entry receptor with the addition of the TGFβR binding peptide, CKS17, to modified oncolytic adenovirus while evading inflammatory macrophage engulfment via IgM binding to the unmodified adenovirus hexon [49]. Similar to neuroblastoma, the CSF1R blockade in pancreatic cancers inhibited macrophage infiltration and activated the remaining macrophages into inflammatory antigen-presenting subtype (decreased CD206, PD1, PDL2), resulting in T cell activation and synergistic tumor progression inhibition with immune checkpoint inhibition [50]. GM-CSF-expressing oHSV infection of pancreatic carcinomas had a dose-dependent anti-tumor effect and activated inflammatory macrophages [51]. Taken together, these findings suggest that in different tumor types, the polarization of inflammatory or immunosuppressive macrophages can enhance oncolytic virus therapy through anti-tumor immune activation or enhanced oncolysis, respectively.

## 4. Conclusions

**Figure 1:**
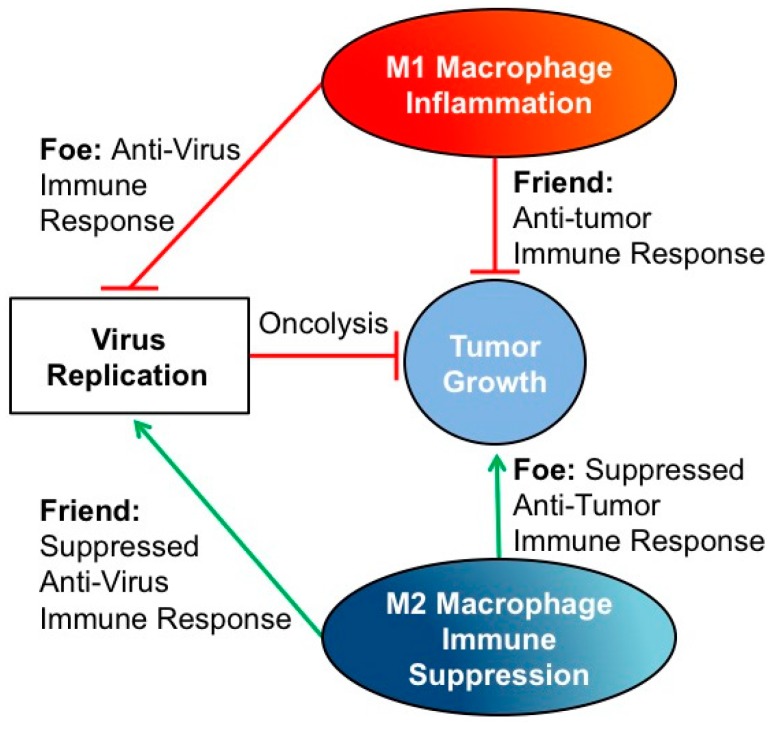
Influence of tumor-associated macrophages on oncolytic virotherapy. In general, although M1-like macrophages may lead to enhanced virus clearance, they appear to be a friend in terms of therapeutic effect as they enable anti-tumor immunity. In contrast, M2-like macrophages tend to be a foe as they enhance tumor growth and immunosuppression. There are exceptions to these generalizations, such as in glioblastoma, breast cancer, and pancreatic cancer where there is evidence of the opposite effects. There are thus likely as yet unknown factors unique to each tumor type or perhaps even to different individuals that influence the direction and impact of macrophages on virotherapy.

While macrophages are generally correlated with poor prognosis in cancer patients, the role of macrophages on oncolytic virus anti-tumor efficacy varies between different tumor models. In most cases, M2-like macrophages seem to be a foe as they may promote tumor growth which trumps any effect on preventing immune clearance of the virus (Figure 1). In contrast, M1-like macrophages are most often a friend, despite their potential for enhancing virus clearance, as they also promote tumor shrinkage (Figure 1). Despite the similarities in macrophage activity between adult and pediatric tumors and an abundance of research supporting macrophage modulation in adult oncolytic virus therapy, there are a limited number of studies that investigate the benefits of macrophage modulation in pediatric oncolytic virus therapy. Some of the few studies investigating oncolytic herpes simplex virus in Ewing sarcoma have shown virus infection induces TNFα and IFNβ in the tumor microenvironment, which have been shown to synergistically kill Ewing sarcoma cells [60]. Oncolytic herpes simplex virus–resistant Ewing sarcoma tumors that express IL-1β, IL-6, and CXCL1 induce CD11b+ cell expression of VEGF after virus infection [28]. Therefore, macrophages may play a significant role in oHSV resistance in Ewing sarcoma because macrophages are the predominant CD11b+ population in the majority of tumor models [10,16]. These studies suggest macrophages likely influence oncolytic virus therapy in pediatric tumors, but further investigation is required in different models to determine if macrophages are friend or foe.

## Figures and Tables

**Table 1:** Summary of macrophage friend or foe outcomes. While tumor-associated macrophages are generally associated with poor prognosis in human patients, the effect of tumor macrophages on oncolytic virus immunotherapy varies by the tumor type and the applied oncolytic virus vector. Despite similarities in the tumor macrophage effect on tumor progression between pediatric and adult cancers, there is a paucity of research investigating macrophage modulation with oncolytic virus immunotherapy in pediatric cancers.

Tumor Type	Macrophage Polarization	Friend or Foe?	Oncolytic Viruses Tested	Signaling Pathways Involved	Reference Number
Osteosarcoma	M2	Foe	none	RAGE, CD24, NfκB, VEGF, MCP-1, HMGB1, IL-10, pSTAT3	[14,20,21,22,25,26,27,28]
Ewing Sarcoma	M2	Foe	none	EWS/FLI1, STAT3, MMP-2, CCND-2, VEGF, MCP-1, M-CSF, RANKL, TNFα, IL-1, VEGF	[22,23,25,26,27,28,29,30,31,32,33,34]
Neuroblastoma	M1	Friend	none	MYCN	[24,35]
-	M2	Foe	none	MIF, Cyclo-oxygenase–prostaglandin E2 pathway, M-CSF	[22,35,36,37]
Colorectal Cancer	M1	Friend	vaccinia virus	GCP-2, KC/GROα, IFNγ, CXCL10, IL-3, IL-6, Lymphotactin, M-CSF1, MIP-1 beta, MCP-1, MCP-3, MCP-5, RANTES, macrophage metallelastase	[38,39,40]
Glioblastoma	M1	Foe	herpes simplex virus	TNFα, CCN1, IL-1β, IFNγ, CXCL10, MCP-1, MCP-3	[41,42,43,44]
-	M2	Friend	herpes simplex virus	TGFβ	[45]
Breast Cancer	M1	Friend	paramyxovirus	Human Monocyte-Derived	[46]
-	M2	Foe	adenovirus	TGFβ	[47]
-	M1	Foe	vesicular stomatitis virus	JAK/STAT, IFNα, IFNβ	[48]
Pancreatic Cancer	M2	Friend	adenovirus	TGFβR, TGFβ	[49]
-	-	-	herpes simplex virus	Nectin-1, TGFβ	[41]
-	M2	Foe	herpes simplex virus	CSF1R	[50]
-	M1	Friend	herpes simplex virus	GM-CSF	[51]
